# Rapid method for the high purity isolation of bovine milk-derived extracellular vesicles via polyester (PET) capillary-channeled polymer (C-CP) fiber columns

**DOI:** 10.7150/ntno.129284

**Published:** 2026-01-30

**Authors:** Carolina Mata, R. Kenneth Marcus

**Affiliations:** Department of Chemistry, Biosystems Research Complex, Clemson University, Clemson, SC 29634-0973, USA.

**Keywords:** bovine milk-derived extracellular vesicles, PET C-CP fiber column, rapid EV isolation, hydrophobic interaction chromatography

## Abstract

The use of raw bovine milk as a source of extracellular vesicles (EVs) has gained in interest for therapeutic applications due to its low cost, accessibility, low immunogenicity, and potential for oral delivery. To address the need to achieve higher throughput isolation of high-quality EVs, high performance liquid chromatography (HPLC) using a hydrophobic interaction chromatography (HIC) capture/elution program is performed on microbore, capillary-channeled polymer (C-CP) fiber columns.

**Methods:** Bovine milk is first skimmed to reduce the fat-laden matrix, followed by treatment using acetic acid (Ac) to precipitate casein micelles before isolation of milk-derived EVs (MDEVs) via HPLC using an HIC process modality with PET C-CP fiber columns. The treated milk is introduced to the column under EV binding conditions, where an ionic solvent with a small amount of organic modifier causes salts, small molecules, and proteinaceous species to pass through unretained while retaining the EVs on-column. The target EVs are eluted by decreasing the ionic strength of the solvent and increasing the elution strength.

**Results:** EVs isolated using PET C-CP fiber columns demonstrate the removal of >95% of matrix-related concomitant species and yield particle densities on the order of 10^11^ particles mL^-1^ in 20 min. Validation of the success of the separation is demonstrated through response curves, nanoflow cytometry, transmission electron microscopy, and protein assays in accordance with MISEV guidelines.

**Conclusions:** A rapid approach to the high yield isolation of high-quality MDEVs via microbore-scale PET C-CP fiber columns is presented here. Each separation yields MDEVs on the order of 4 x 10^11^ particles mL^-1^, in 20-min at a cost of <$5 per column. Paths forward to greater EV throughput and yields are currently under development.

## 1. Introduction

Extracellular vesicles (EVs) are membrane bound bionanoparticles that include a subpopulation, exosomes, which range in size from 30-200 nm 1. EVs are excreted from all cell types through a variety of pathways into the extracellular space where they participate in cell signaling and communication. Additionally, their innate ability to carry and transport cargo including proteins, RNA, and DNA to particular cells makes them especially promising as transporters of therapeutics 1-3. EVs employed as therapeutic vectors are sourced from a variety of biofluids, as well as cell culture supernatant and apoplastic wash from plants. Despite their ready availability, their eventual application potential depends on the total concentration and level of purity of the isolates, i.e. how well they are isolated from host matrix-related species. Effective isolation methods are imperative as therapeutic administration methods differ, with the need for concentrations of EVs varying significantly (10^6^-10^11^ particles mL^-1^) 2, 4, 5, with the general purity standard for EV isolates being 3.0×10^10^ particles µg^-1^ of latent protein 6. Potential vectors would be harvested with this target, importantly having a lack of immunogenicity, maintaining bioactivity, and having the capacity for cargo modification 2, 6.

To address the need for high quality EVs, bovine milk is proposed as a source for therapeutics as it is widely available, collected non-invasively, is an inexpensive-product, and is enriched with EVs at levels of 10^6^-10^11^ EVs mL^-1^ 2, 4, 5, 7, 8. Milk-derived extracellular vesicles (MDEVs) are known to be biocompatible across species, ensuring they do not contribute to detrimental immune or inflammatory responses, and resist denaturation from temperature, pH fluctuations, and enzymatic processes 2, 4, 9-13. Resistance to environmental changes (including within the gut) makes MDEVs prime candidates for orally delivered therapeutics, there have been a variety of studies where MDEVs implemented as vectors exhibit promising results 2, 4, 9-13. While the future of MDEVs as therapeutic vectors is promising, the incredibly complex matrix of bovine milk, including fats, lipids, and proteins, is itself a major hindrance to the effective isolation of the desired EVs 8, 14, 15.

Complex matrix effects are not uncommon across the field of EV isolation methods, whether the source is plasma/serum, cell culture media, or plant-based matrices, with the suite of the more common isolation methods itself being very diverse 16. Each matrix type poses its own set of challenges, but those of bovine milk are raised to another level as high milk-fat and protein content require extensive pre-processing, regardless of the EV isolation methodology. Fortuitously, standard skimming processes are effective for fat removal. More challenging, the high concentrations of proteins such as casein, which forms micelles that mimic the target MDEV sizes, hydrophobicity, and density, pose hurdles prior to attempting standard EV isolation protocols 9, 11, 13, 17. Efforts to minimize casein effects on MDEV isolation include cloud point precipitation, ultracentrifugation and the addition of sodium citrate, rennet, or other acidic media for precipitation, with acid precipitation being the most commonly employed 17-20.

Once the high-level protein extraction has been affected, MDEV isolation methods including ultracentrifugation (UC), tangential field flow filtration (TFF), asymmetric-flow field-flow fractionation (AF4), size exclusion chromatography (SEC), free-flow isoelectric focusing (FFIEF), and polymer precipitation struggle to meet the same concentration and purity standards they meet with the less complex matrices 4, 8, 11, 13, 14, 21-29. UC separates EVs and matrix components based on a density gradient, and while effective in sedimentation of heavier matrix components, less dense species including EVs, microvesicles, low density lipoproteins (LDLs), and cellular debris are co-sedimented as centrifugal force increases 21, 22. Work by Ye et al. has demonstrated alternate routes to achieving the scalable isolation of EVs through the pre-treatment and pre-concentration of bovine milk before isolation via UC 30. Using this workflow, milk is pre-treated by enzyme-assisted precipitation before concentrating with a molecular weight cut-off membrane for UC isolation 30. While effective in terms of vesicle concentration, further scaling of the separation platform itself remains a challenge as the equipment itself is very expensive and ultimately limited in practical sizing.

Commercial polymer precipitation kits can be effective to process small sample volumes (single mL). However, with coprecipitation of other constituents, the overall poor solubility of EVs, and very limited throughput, further downstream analysis/utilization is difficult. Gravity fed SEC, being a size-based method, elutes EVs along with other matrix components of similar size, especially LDLs, and so isolate purities are sacrificed 22, 31. Another size-based separation, TFF offers the bulk separation of EVs by flowing a solvent tangentially across a membrane having the desired pore sizes, allowing the separation to be customized for concentration/purity 11, 25. A constantly flowing solvent minimizes membrane fouling, but increases shear stress on vesicle membranes and impacts final EV concentrations via dilution 22, 25. A subclass of field flow fractionation, AF4, introduces carrier buffers through two channels perpendicular to each other. Here, the channel perpendicular to the forward flow pushes the smaller molecular species toward a porous membrane for elution 26, 27. Coelution of EVs with similarly-sized matrix-related species (e.g., protein aggregates) is commonplace, and bulk isolations are a challenge, as only small sample amounts (100 µg) can be processed at a time 26, 27. FFIEF affects an electrochemical separation of EVs by flowing carrier buffer through a channel with a perpendicular pH gradient. When voltage is applied, the EVs migrate until they reach their isoelectric point 28. Khan et al. have developed a reciprocating FFIEF method for the large-scale isolation of EVs, where the carrier solvent is flowed rapidly to mitigate the previous limitations of gravitational and electrical convection effects 28, 32.

An alternative novel and straightforward EV isolation method exists in the form of polymeric fiber chromatographic platforms. Marcus et al. have developed polyester (PET) capillary-channeled polymer (C-CP) fiber columns and spin-down tips for the isolation of EVs from diverse matrices 16, 33, 34. High performance liquid chromatography (HPLC) columns are assembled by pulling the melt-extruded eight pronged (PET-8) or three-pronged (PET-Y) polyester fibers through 0.8 mm diameter and 30 cm long, polyetheretherketone (PEEK) tubing, termed a microbore column format 33. PET-8 and PET-Y fiber columns differ only in geometry, and therefore surface area, while maintaining the same surface chemistries, with the former allowing for increased stationary phase and analyte interaction 35. Use of the microbore column format allows ready implementation on standard HPLC systems, with operation at very low backpressures (<500 psi) and high volume throughput. Spin-down tips are assembled similarly using 0.8 mm diameter, 1 cm long fluorinated ethylene-propylene (FEP) tubing. The columns are affixed to standard 200 μL low-retention micropipette tips for multi-sample processing in parallel on a benchtop microcentrifuge 34. Successful isolation of high purity EVs has been achieved from a plethora of complex matrices including urine 36, 37, plant apoplastic washes 38, cell culture supernatants 37, 39, 40, blood plasma 41, and most recently, bovine milk 42.

The initial efforts towards MDEV isolation on the C-CP fiber platform took from the previously-cited works wherein the skimming and acidification of the raw milk were employed before injection onto the PET-8 fiber columns 42. Briefly, raw bovine milk was skimmed, diluted 1:1 with 1× phosphate buffered saline (PBS) and precipitated with 6% v/v acetic acid (Ac) 43, 44. After centrifugation and discarding the precipitant, the treated milk was filtered with a 0.22 µm polyethersulfone (PES) syringe filter 45, and 100 µL were injected on to the fiber column for isolation via hydrophobic interaction chromatography (HIC). After this pre-treatment, PET-8 C-CP fiber columns affected the isolation of MDEVs from the complex matrix with ease. A western blot immunoassay of the lysed MDEV eluate determined more than 80% of the total casein was effectively removed through a combination of Ac pre-treatment of bovine milk and isolation via the fiber columns 42, 46. This method yielded high purity MDEVs with particle counts on the order of 1.5×10^10^ particles mL^-1^ with corresponding purities of ~2.0×10^10^ particles µg^-1^ protein. While a purity standard and reference material for MDEVs are yet to be established, the current standard for human biofluid-derived EVs is 3.0×10^10^ particles µg^-1^ protein 6. However, this standard does not take into consideration the difference in concentration of vesicle-associated proteins between human biofluids and bovine milk; MDEVs have over 2000 associated surface proteins that also contribute to the “total protein” content in the Bradford-type assays, leading to an underestimation of the true purity of the isolates 7, 46, 47. Despite the lack of a standardized purity value for this matrix, EVs isolated with PET C-CP fiber columns were within proximity of purity expected from less complex samples, speaking to the efficacy of the isolation method.

Here, with the end goal of achieving high-yield MDEV isolations for therapeutic applications, initial characterization of the processing aspects of the trilobal PET-Y C-CP fiber columns (albeit on the microbore scale here) towards preparative scales was undertaken. Using the previously optimized protocol for milk Ac pre-treatment 42, the dynamic binding capacity (DBC) of the columns towards MDEVs was evaluated via frontal analysis of Ac-treated raw bovine milk. By directly infusing the Ac-precipitated milk onto the column under EV binding conditions, 1M ammonium sulfate (AMS) and 20% acetonitrile (ACN), the amount of EVs recovered increased by 10x in comparison to the previous 100 µL injections used in the “analytical” determinations 42, while maintaining comparable purities. Characterization of MDEVs isolated via PET-Y C-CP fiber columns was performed with consideration of minimal information for studies of extracellular vesicles (MISEV) standards 23. Characterization included verification of physical structure via transmission electron microscopy (TEM), immunoconfirmation using two distinct tetraspanin fluorescent labels and a lipophilic membrane dye via nano-flow cytometry, purity assessments using standard response curves and protein assays, and quantification via optical absorbance. The recovery of MDEVs from Ac-treated milk via chromatographic bind-and-elution from PET-Y C-CP fiber columns holds promise for the bulk recovery of MDEVs and application in downstream therapeutic practices, with efforts towards even greater throughput currently under development.

## 2. Methods

### 2.1 Instrumentation

A Thermofisher Scientific Dionex Ultimate 3000 HPLC equipped with an LGP-3400SD quaternary pump and MWD-3000 UV-Vis absorbance detector with a 13 µL flow cell (Thermo Fisher Scientific, Sunnyvale, CA, USA) was used to perform the isolations. Detection occurred at 216 nm, and the instrument was controlled by Chromeleon 7 software. A second instrument was used for quantification of EVs; a Thermo Fisher Scientific Vanquish Flex system (Sunnyvale, CA, USA) equipped with an autosampler (split sampler F), quaternary pump (Quar. Pump F), and a UV-Vis photodiode array detector (diode array detector FG) with a 13 µL biocompatible flow cell. The system was controlled using Chromeleon 7.3.2 software. To skim bovine milk and sediment precipitants in preparation for separation, a VWR symphony 4417 tabletop centrifuge (Rador, PA, USA) was used. An Agilent BioTek Synergy LX Multi-Mode Plate Reader (Santa Clara, CA, USA) was used for Bradford protein assay detection at 595 nm. The NanoFCM Nanoanalyzer (Nottingham, Nottinghamshire, UK) was employed to determine particle size and concentrations, with fluorescence detection used for immunoconfirmation. Images of vesicles were captured using a JEOL 2100PLUS microscope housed in UGA's Georgia Electron Microscopy facility.

### 2.2 Chemicals and reagents

Chemicals used to make solvents for HIC isolation including AMS and ACN were purchased from VWR (Sokon, OH, USA). PBS (Gibco pH 7.4) and glacial Ac were purchased from ThermoFisher Scientific (Waltham, MA, USA). All HPLC solvents prepared used deionized (DI) water from an Elga PURELAB flex water purification system (18.2Ω cm^-1^) (Veolia Water Technologies, High Wycombe, England). Bradford Pierce Coomassie Plus Reagent^TM^ was purchased from Thermo Fisher Scientific (Waltham, MA, USA). Due to a lack of available MDEV reference materials, but similarities in matrix related protein content, lyophilized human embryonic kidney (HEK) EV standards (3.7×10^11^ particles mL^-1^) purchased from Galen Molecular (North Haven, CT, USA) were used to generate optical absorbance response curves. Standards for NanoFCM calibration (Nottingham, Nottinghamshire, UK) included 0.25 µm fluorescent silica microspheres (QC Beads) with a particle count of 2.17×10^10^ particles mL^-1^, and a cocktail of silica nanoparticles (S16M-Exo) in 4 different size populations (68, 91, 113, and 155 nm). Immunolabeling of vesicles was accomplished using anti-CD81 and anti-CD9 fluorescent antibodies (Abs) (1:2000 final dilution) and were purchased from Biotium (Freemont, CA, USA). The Memglow lipophilic membrane dye (1:4000 final dilution) was purchased from Cytoskeleton Inc. (Denver, CO, USA).

### 2.3 Precipitation of raw bovine milk

Raw Holstein bovine milk for MDEV isolations was provided by Clemson University's LaMaster Dairy Piedmont Research and Education Center. Milk was collected using a Delaval V300 milking station (Tumba, Sweden) and decanted into a 1 L glass solvent bottle for transportation to the laboratory (~5 min). Milk was aliquoted into 15 mL falcon tubes (VWR, Sokon, OH, USA); the majority were stored at -20ºC for short term storage (1-2 weeks) and 2 were stored at 4ºC for immediate pre-treatment and MDEV isolation. To deconvolute the complex milk matrix, the raw milk was skimmed (4,180×g, maximum centrifuge speed) and diluted 1:1 with 1×PBS. Skimmed and diluted milk was precipitated using 6% v/v glacial Ac and incubated on benchtop for 5-min 42, 43. The precipitated milk was then centrifuged at 4,180×g for 10 min before discarding the precipitant and filtering the supernatant with 0.22 µm PES syringe filters (FroggaBio, Toronto, Canada).

### 2.4 PET C-CP fiber column preparation

The PET-Y C-CP fibers employed in the separation columns were melt-extruded by Universal Fibers, Inc. (Bristol VA), with the microbore fiber column assembled as previously described 36. Here, four rotations (264 fibers) of PET-Y (trilobal/Y-shaped) fibers were heat shrunk using hot DI water before being packed into PEEK tubing (30 cm length and 0.76 mm in diameter). Fiber interdigitation upon feeding the fiber bundle through the PEEK tubing creates 1-5 µm wide channels where the EVs pass and interact with the stationary phase. The interstitial fraction (*ε_i_*) of 4-rotation PET-Y C-CP fiber columns was 0.69 ± 0.03 35. Previously, PET-8 (eight lobed) fibers were used for separations, however recent experiments demonstrating the increased dynamic binding capacities of four rotations of PET-Y provided increased available surface area to maximize EV adsorption 35. With the end goal of high yield separations of MDEVs for therapeutics, PET-Y fiber columns were implemented here. These columns are optimized for use in the HIC modality 33.

### 2.5 HIC chromatographic method

The general HIC separation method developed for a wide variety of matrices has been followed for the isolation of MDEVs 33, 42. In the method, the sample is loaded under high ionic strength solvent conditions (2M AMS) allowing for salts, small molecules, and generic materials to pass, while more hydrophobic matrix proteins and EVs are retained. In the first gradient step, those hydrophobic proteins are eluted when the ionic strength is decreased and a small amount of organic modifier is added, while EVs are retained on fiber. Finally, the most-hydrophobic EVs are eluted by significantly reducing the mobile phase ionic strength and increasing the organic solvent strength 33. The dynamic binding capacity towards EV isolation used here was adapted from the frontal loading method developed for urine- and HEK culture-derived EVs on to PET C-CP fiber columns 35, 37. The skimmed and diluted milk was treated with 6% v/v Ac for protein precipitation before separation 42. Here, the PET C-CP column is equilibrated in 1M AMS and 20% ACN (EV binding conditions) at a flow rate of 0.5 mL min^-1^ for 3 min. The endpoint of the equilibration is determined by a stable (flat) baseline absorbance reading at 216 nm. The 20-min isolation begins with the frontal loading of Ac-treated milk via solvent line A, mixed 1:1 with EV binding solvent (final loading concentration 0.5M AMS and 10% ACN) for 2-min. The loading phase is followed by a 10-min wash with the same 1M AMS and 20% ACN buffer. MDEV elution is affected in 40% ACN (balance PBS) with the EV-containing fraction passing as a band in <5 min. The loading and EV elution fractions were collected manually for evaporation of 40% ACN overnight at 5ºC before quantification 33. Multiple previous studies have revealed no ill effects in the use of this isolation methodology with regard to vesicular integrity or surface protein coverage.

### 2.6 Validation of vesicular structure

Verification of intact vesicular structures was accomplished post-isolation via transmission electron microscopy (TEM). TEM images were taken using a JEOL 2100PLUS microscope using a uranyl acetate negative staining method as detailed by Jung et al. with some adjustments 48. Copper carbon-coated grids (Electron Microscopy Sciences, PA, USA) were incubated twice with 10 µL MDEV isolates for 5-min, excess sample was wicked off using filter paper after each incubation. The grid was subsequently washed by dabbing it face down on top of a drop of DI for 2-3 sec, then wicking dry. The grids were then placed upside down on top of a 2% paraformaldehyde drop (16% stock concentration #15710 Electron Microscopy Sciences, PA, USA) for 5-min before being transferred to 2 drops of sterile PBS, wicking dry in between drops. Grids were placed upside down on a final PBS droplet and allowed to sit for 2-min, after being wicked dry the grid was rested on a drop of 1% uranyl acetate (Electron Microscopy Sciences, PA, USA) for 15-sec before being wicked dry. Grids were then stored in the vacuum desiccator for ~30 min to dry before imaging.

### 2.7 Size distribution, particle count, and immunoconfirmation

The NanoFCM Nanoanalyzer (Nottingham, Nottinghamshire, UK) is a nanoflow cytometer that has been adapted for single particle analysis of bionanoparticles. This instrument is equipped with two lasers and several bandpass filters for optical detection in three channels: side scattering (bandpass filter: 488/10 nm), green fluorescence (bandpass filter: 525/40 nm), and red fluorescence (bandpass filter: 670/30 nm). The side scatter channel is used for the size and concentration estimates based on the scattering of EV particles, while the fluorescent channels allow detection of up to two unique fluorophores simultaneously. Calibration of the instrument before each use followed the manufacture guidance, where 1:99 dilutions in DI water of QC Beads and S16M-Exo sizing beads are used. All solvents were filtered using a 0.22 µm polytetrafluoroethylene (PTFE) hydrophilic syringe filter as recommended by the manufacturer. Instrumentation blanks were acquired via introduction of: EV isolates that were not fluorescently tagged, neat 1×PBS, and label-added 1×PBS. MDEV fractions had 40% ACN evaporated off overnight at 5ºC and diluted to ~2.0×10⁸ particles mL⁻¹ with 1×PBS. These fractions were labeled with fluorescent anti-CD81 Abs (1:2000 final dilution), anti-CD9 Abs (1:2000 final dilution), and lipophilic membrane dye (1:4000 final dilution) before incubation in a 37ºC water bath protected from light for 2-h. Samples were introduced to the NanoFCM Nanoanalyzer according to manufacturer instructions, and detected using the side scatter, FITC (490 nm excitation and 516 nm emission), and PE-Cy5 (630 nm excitation and 680 nm emission) channels. Data was processed using the NF Profession 2.3 software.

### 2.8 Particle quantification

Post column optical absorbance has been shown to be an effective means of EV particle concentration determinations 36. Total particle concentration of EVs was determined using a Thermofisher Vanquish HPLC with a 50 µL loop in column bypass mode, and detection via absorbance at 216 nm. In the absence of a true standard reference material, an aliquot of the previously mentioned HEK standard (3.7×10^11^ particles mL^-1^) was injected 2 µL at a time at 0.5 mL min^-1^ before successive 1:1 serial dilutions with 1×PBS. The response curve concentration range spanned 2.31×10^10^ - 3.70×10^11^ particles mL^-1^. Each standard dilution was injected in triplicate (n=3), with a Beer's Law correlation of R^2^= 0.98. MDEV fractions were injected in 2 µL aliquots in triplicate (n=3).

### 2.9Protein quantification

To determine the purity of MDEV eluates from the Ac-treated raw milk, a Thermo Fisher Scientific Coomassie Bradford Protein Assay Kit was employed. Fractions from the HPLC separation were collected in 500 µL aliquots from the EV elution fractions. Fractions were stored overnight at 5ºC for ACN evaporation, leaving them in a 1×PBS solution and brought back to the original 500 µL collection volume with 1×PBS before analysis. Bovine serum albumin (BSA) 2 mg mL^-1^ was used as the stock solution as per manufacturer instructions for standard curve generation instead of EV 'standards' due to its dynamic concentration range (25 µg mL^-1^-2 mg mL^-1^) and the questionable purity of those materials. For sample application on to a 96-well plate, 5 µL of each standard and sample were applied in triplicate before adding 250 µL of the Pierce Coomassie Bradford Assay reagent. The plate was agitated for 30-sec in the Agilent BioTek Synergy LX Multi-Mode Plate Reader (Santa Clara, CA, USA) and then incubated on benchtop for 10-min before triplicate absorbance readings at 595 nm (n=3).

## 3. Results and Discussion

### 3.1 MDEV isolation methodology

The primary goal of the described effort is the development of a straightforward, efficient, and reproducible chromatographic method for the isolation of MDEVs. Here, the targets include the amount of captured and recovered EVs and the utilization of the fiber column surface area.

#### 3.1.1 Chromatographic method development

**Figure 1:**
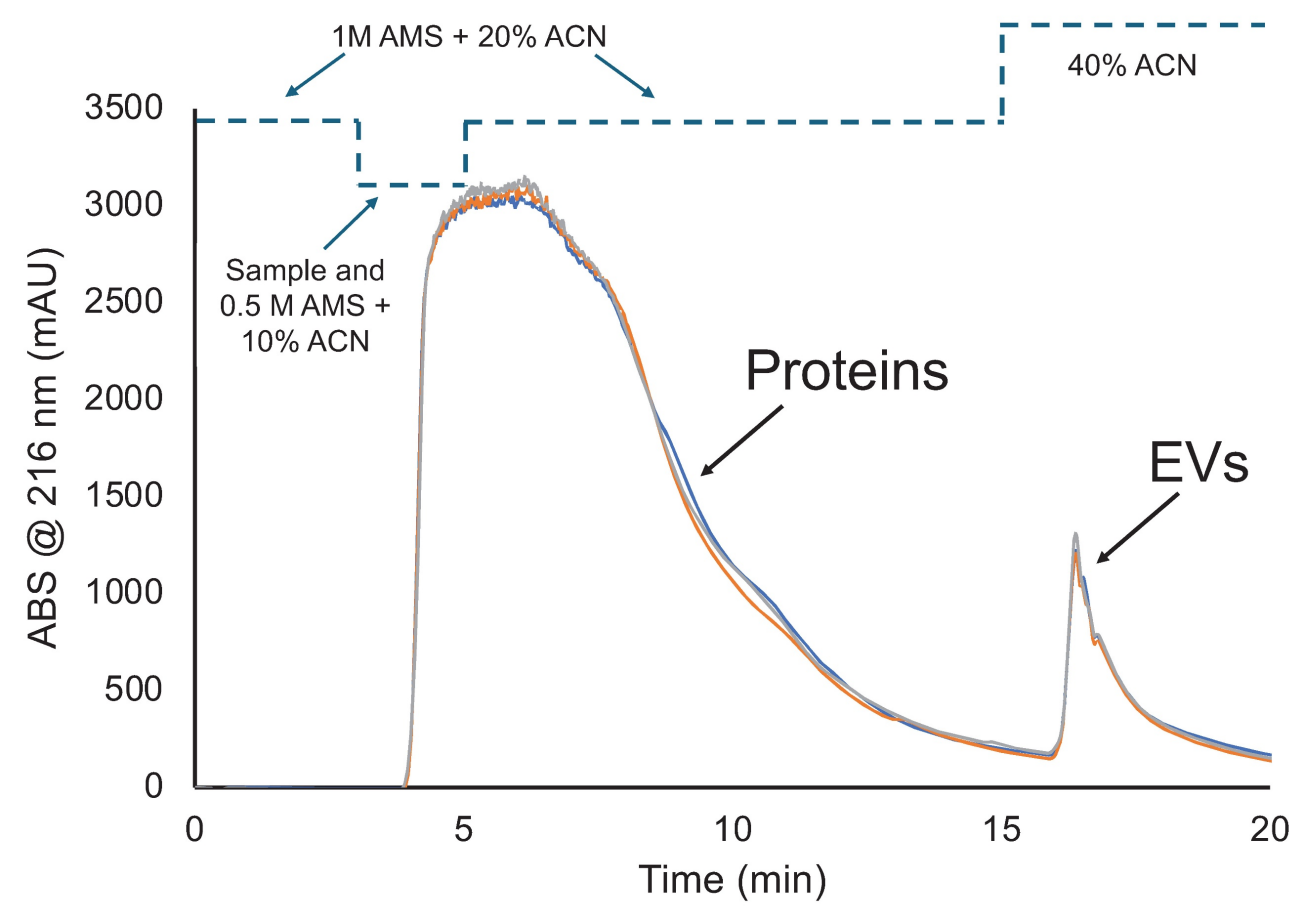
HIC chromatogram demonstrating triplicate isolations of MDEVs after frontal loading. Representative signals include the initial rapid saturation of the PET C-CP fiber column and a second transient represents the recovered MDEVs.

This chromatographic method for the determination of dynamic binding capacity of EVs was developed based on past HIC frontal loading studies of urine and HEK cell culture supernatants 35, 37. **Figure 1** demonstrates a typical frontal loading experiment, inclusive of bind-and-elution steps, for triplicate injections of the skimmed, Ac-treated bovine milk. The general concept is to introduce the test matrix to the column under solvent conditions wherein the target EVs are retained while other matrix components pass, termed EV binding conditions. Here 1M AMS and 20% ACN act as both the loading and washing phases, where proteins/lipoproteins and other nominally hydrophobic species pass through or are released from the fiber phase. As seen in the temporal program, the fiber column was first equilibrated in 1M AMS and 20% ACN at 0.5 mL min^-1^, with the Ac-treated milk introduced 1:1 with the same solvent for a final loading buffer concentration of 0.5 M AMS and 10% ACN at t = 3 min. Under continuous introduction of Ac-treated milk/loading buffer solution, the column becomes saturated to the point where no further EV/matrix species are retained and “breakthrough” is observed with the onset of absorbance (or scatter in the case of EVs) at 216 nm. (Note, the transit times for the various changes in solvent fronts is ~2.5-min.) The steep breakthrough slope plateaus within 1.5 min of onset of the sample loading, indicative of column saturation. Following the onset of the plateau, the column is washed for 10-min using the same EV binding buffer to minimize protein carryover in the MDEV elution fraction. The slow return to baseline between loading and EV elution indicates the success of the wash in limiting protein/lipoprotein carryover. For MDEV elution and fraction collection, a 5-min 40% ACN in 1×PBS step is initiated (t=15-min) and a second signal transient is observed denoting elution of MDEVs. The return of the signal to baseline (t=20-min) denotes the end of the method. The repeatability of the capture-elution process, inclusive of complete saturation of the fiber phase is clearly demonstrated in the proximity of the three chromatograms. Most impressively, is the quantitative recovery demonstrated in the MDEV elution bands, where the triplicate experiments yield average peak areas of ~940 mAU×min with a variability of <1 %, relative standard deviation (RSD). This level of consistency speaks to the lack of appreciable fouling or carryover between the isolation cycles. While not evaluated quantitatively here, replicates of the DBC studies of PET C-CP fiber columns suggest that 6 complete saturation cycles can be performed with no significant degradation of recovery observed.

#### 3.1.2 EV quantification and dynamic binding capacity

There are two aspects of characterizing the efficiency of EV isolation and recovery. In the first case, the concentration of EVs in the load solution (EVs mL^-1^) is paired with the solution flow rate (mL min^-1^) and the time (min) corresponding to column saturation, to yield a number of adsorbed particles. In the second case, the area of the EV elution peak is correlated with a separately-generated response function via Beer's law 36. To be clear, the quantification of EVs of all forms is complicated by the fact that there are no standard/certified reference materials (SRM/CRMs) which provide purely-isolated EVs of known absolute number density. As such, “standards” of uncertain purity and density must be used. The number density of MDEVs in the bovine milk matrix was determined by the method of standard addition of the HEK-originating standard to the Ac-treated sample. The increased responses upon addition of the standard were used to back-calculate the initial concentration, which was determined to be 1.85 ×10^12^ particles mL^-1,^ with a <4 % relative deviation among triplicate measurements. Prior to the quantification of the EV elution peak (recovery), the ACN in the collected fractions from HIC separations were evaporated overnight at 5ºC. An absorbance-based response curve was constructed by injecting 2 µL aliquots of serial dilutions (in 1×PBS) of the HEK EV primary standard (3.7×10^11^ particles mL^-1^) in triplicate (n=3), yielding a linear response having an R^2^= 0.98, MDEV isolate fractions from Ac-treated milk separations were injected in the same fashion (n=3), yielding concentrations of 4.0×10^11^ particles mL^-1^ with a <1% RSD observed.

Methods for the determination of dynamic binding capacity (DBC) have been previously described 35, 37. In this case, DBC determination towards MDEVs was calculated using the amount of particles loaded on to the column (1.85×10^12^ particles mL^-1^), a flow rate (0.5 mL min^-1^), the load-time to reach the 50% column saturation via absorbance (t_avg_ = 1.16-min), and column surface area (0.011 m^2^) 35. The dynamic binding capacity based on the frontal loading was determined to be 1.95×10^14^ particles m^-2^ , with a <1% RSD, a value that is comparable with previously reported DBC, 1.22×10^14^ particles m^-2^ found for PET-Y C-CP fiber columns saturated with urine derived EVs 35, 37. When comparing the DBC of both MDEVs and urine derived EVs, one must consider this measurement is in part reliant on absorbance (in the case of EVs, scattering) measurements. As Ac-treated milk matrix is much more proteinaceous than that of urine, it is within reason that the DBC reported here may be impacted by contributions of absorbance by free protein. With respect to the practical aspects of EV isolation throughput, the breakthrough data represent an average capture of 1.07 x 10^12^ EVs within each 20-min load/elution cycle on these microbore column structures.

To estimate the surface utilization efficiency of the PET C-CP fiber columns, the area of an average sized EV (100 nm) and total fiber surface area (0.011 m^2^) are taken into account for an estimate of 1.40×10^12^ particles needed to fully saturate the fiber surface under ideal conditions. Previously, the total number of urine derived EVs loaded was 1.32×10^12^ particles, with the surface area utilization efficiency being >90%, however studies using electron or fluorescence microscopy imaging would be needed to verify this level of coverage 35. Here with the Ac-treated milk, the 1.85×10^12^ particles would suggest a surface area utilization of above 130%. Of course, this utilization is also based on the assumption of the singular particle size and the accuracy of the determined MDEV load solution concentration. Here again, microscopic imaging would be required to assess the level of coverage. Likewise, use of more varied loading conditions could allow the construction of adsorption isotherms which would reflect nature of the surface coverage, for example the existence of multilayering 32, 49. As the isolation of MDEVs via PET C-CP fiber columns moves toward scale- up, further investigation involving fiber surface area utilization efficiency is needed.

### 3.2 Characterization of MDEV Isolates

Following the basic characterization of the capture/release of MDEVs on the PET C-CP fiber column, it is obvious that the physical and chemical efficacy of the isolates must be validated. Clearly, successful isolation without retention of basic EV characteristics renders the methodology useless for downstream application of the target vesicles. Here, the generally accepted suite of instrumental methods is used verify the fact that intact EVs are indeed harvested. To be clear, the ultimate demonstration of efficacy would involve in-vivo uptake experiments, but those are only reasonable after evaluations of the sort performed here.

#### 3.2.1 Microscopic vesicular structure confirmation

**Figure 2:**
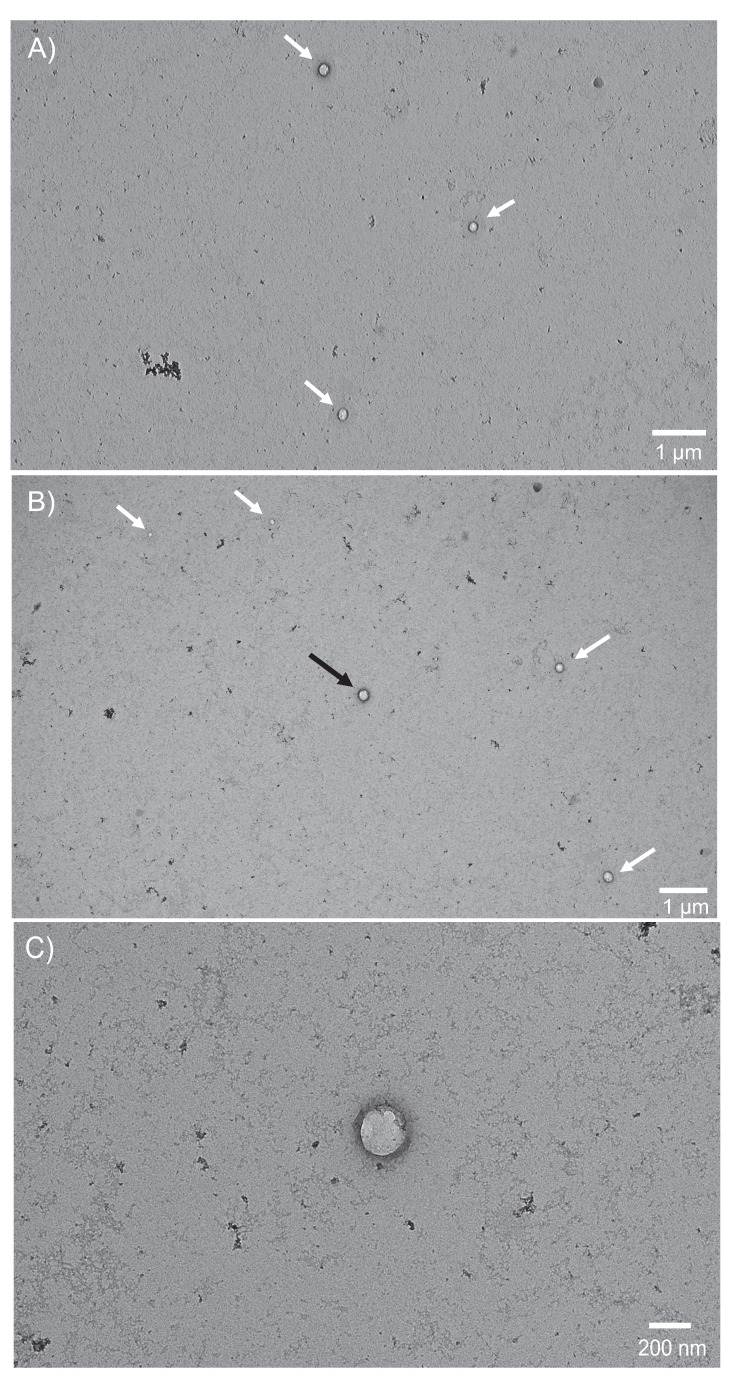
** A-C:** Micrographs captured using a TEM are representative of vesicles isolated from Ac treated bovine milk using a PET-Y C-CP fiber column in A) and B) wide views (1 µm scale) with multiple EVs in frame within the size range of 30-200 nm and C) close-up view (200 nm scale) of the EV located in the center of view B, displaying the distinct round shape and membrane structure inherent to EVs.

Transmission electron microscopy is the gold standard to verify whether or not isolated EVs are in the correct size range and exhibit their fundamental vesicular morphology 23. **Figure 2A** presents a wide-field (~5 x 10 μm) view, wherein 3 MDEVs are highlighted with the white arrows. Round structures with bilayer membranes that are enhanced in the negative staining are observed along with very minor contributions from other matrix related artifacts (such as aggregated uranyl acetate and proteins) 50. In** Fig. 2B**, a second wide-field view micrograph, is representative of another quadrant in the same grid with 5 additional MDEVs indicated by the arrows. Also visible in frame are matrix-related and/or uranyl acetate aggregates. Vesicles observed here appear to range in size from 50-200 nm in diameter, with smaller vesicles observed near the top left of the micrograph and larger vesicles in the middle and right side of the frame. A closer inspection of the central vesicle in **Fig. 2B** (black arrow), on a 200 nm scale, is presented in **Fig. 2C**, demonstrating the expected morphology of an intact vesicle with a bilayer membrane, and shadow around the outer perimeter of the membrane indicating a 3-D structure. Across a broad series of associated micrographs, vesicle populations within 30-200 nm size range having the expected EV morphology are identifiable using this uranyl acetate negative staining TEM method.

#### 3.2.2 Flow cytometry EV characterization

The NanoFCM Nanoanalyzer provides complementary verification for TEM-derived MDEV size approximations (which of course are a limited sampling) along with immunoconfirmation. The side scatter channel with size detection capabilities found the average size of particles was 122.5 ± 29.3 nm with a 2.6% RSD across triplicate samples involving ~2.0×10^7^ particles per data set. Data acquired from the side scatter channel was used to estimate particle counts for labeling. The optimal concentration towards fluorescence labeling and detection for this instrument is ~2.0×10^8^ particles mL^-1^ according to the manufacturer. Instrumentation blanks were acquired by introducing triplicate fractions of unlabeled EVs into the instrument as well as neat 1×PBS solutions and 1×PBS solutions to which the label solution was added. Particles <50 nm were excluded from data sets, as these particles are below the instrument's side scatter limit of detection. Once MDEV fractions were adjusted to the appropriate concentrations, the fluorescent lipophilic membrane dye was incubated with the MDEV fractions, where the dye intercalates to confirm presence of vesicular membrane. To verify that the vesicular species detected are EVs, immunoconfirmation was accomplished through fluorescent labeling of the tetraspanin membrane-bound protein biomarkers 1. Tetraspanins CD81 and CD9 were targeted using the fluorescent antibodies anti-CD81 and anti-CD9 for detection via the 525 nm FITC channel, while the lipophilic membrane dye was detected in the 670 nm PE-Cy5 channel.

**Figure 3:**
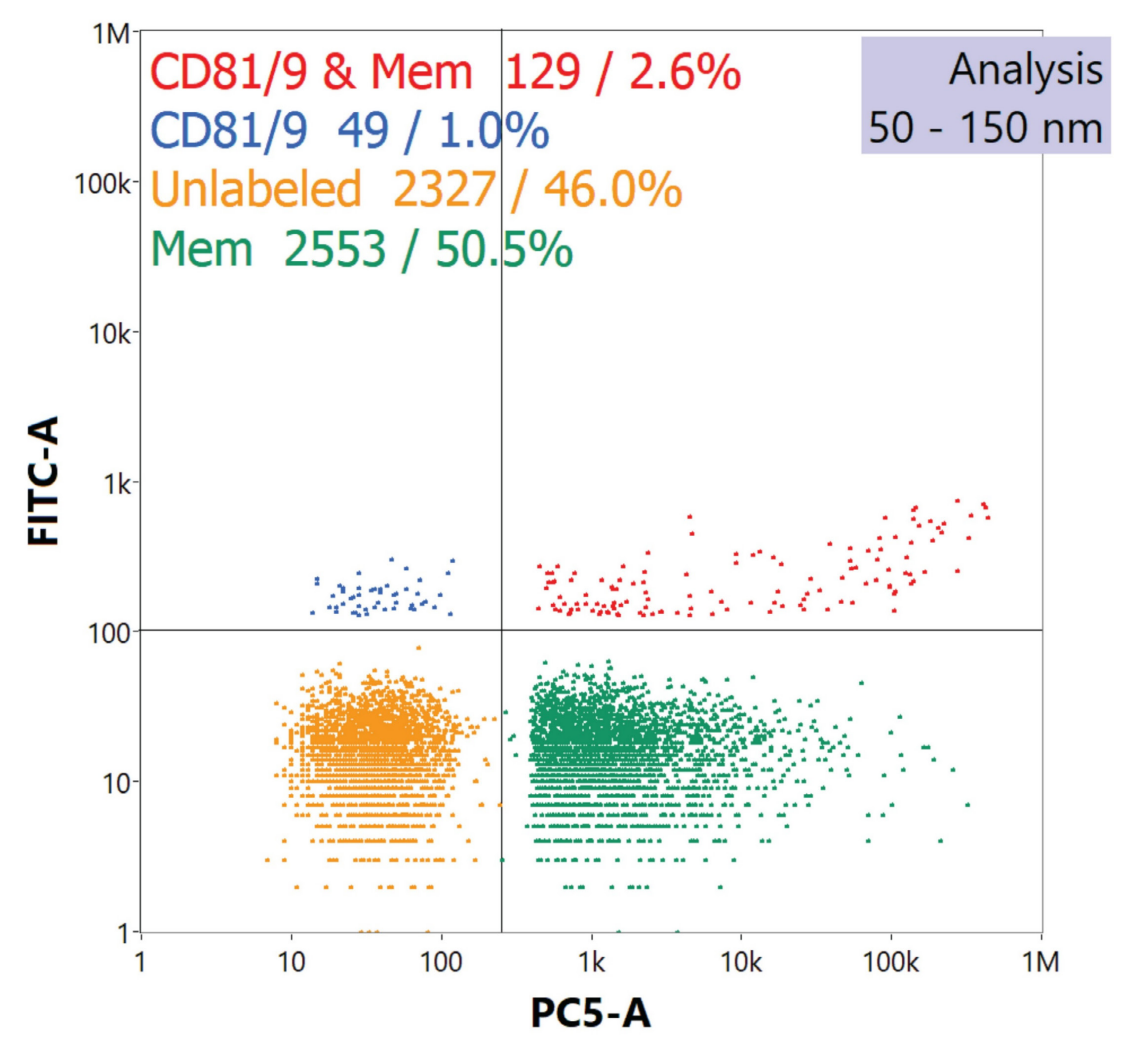
Quad plot generated by nanoflow cytometry demonstrates fluorescent response of isolated EVs after labeling with a fluorescent anti-CD81 and anti-CD9 tetraspanin protein cocktail and a membrane vesicle dye.

A typical reporting to the immunofluorescence assay is presented in the quad chart of **Fig. 3**. The quad plot depicts the relative percentage of particles which register responses towards scattering and the respective fluorescence channels. The bottom left quadrant represents those particle events seen through scattering, but without any corresponding fluorescence signals. The top-left quadrant represents those particles which exhibited both side scattering and fluorescence solely in the channel reflecting the labeling of the tetraspanins. As seen in the legend**,** vesicles labeled with anti-CD81 and/or anti-CD9 Abs yield a 1.0% positive response. This value is not surprising as each EV might be expected to provide only 10-to-15 copies of the surface proteins 51, and so the fluorescence yields for each would be very low. On the other hand, the bottom-right quadrant reflects high-efficiency labelling and detection regarding the membrane-specific dye, where approximately 50% of the particles demonstrate a positive response. Finally, the top-right quadrant reflects the percentage of events wherein both tetraspanin and vesicular membrane dye are coincidently detected. Given the low efficiency towards the former, the cumulative value of 2.6% coverage is not surprising, with this value being in line with previously reported values which were ~3.0% 42. The detection of membrane vesicle dye and EV immunolabels through the NanoFCM Nanoanalyzer indicates that the species isolated were indeed EVs, and that the biomarkers integral for cell-to-cell communication (i.e. cellular uptake) were not likely compromised in either the Ac precipitation or HIC isolation processes.

#### 3.2.3 Determination of EV purity

**Figure 4:**
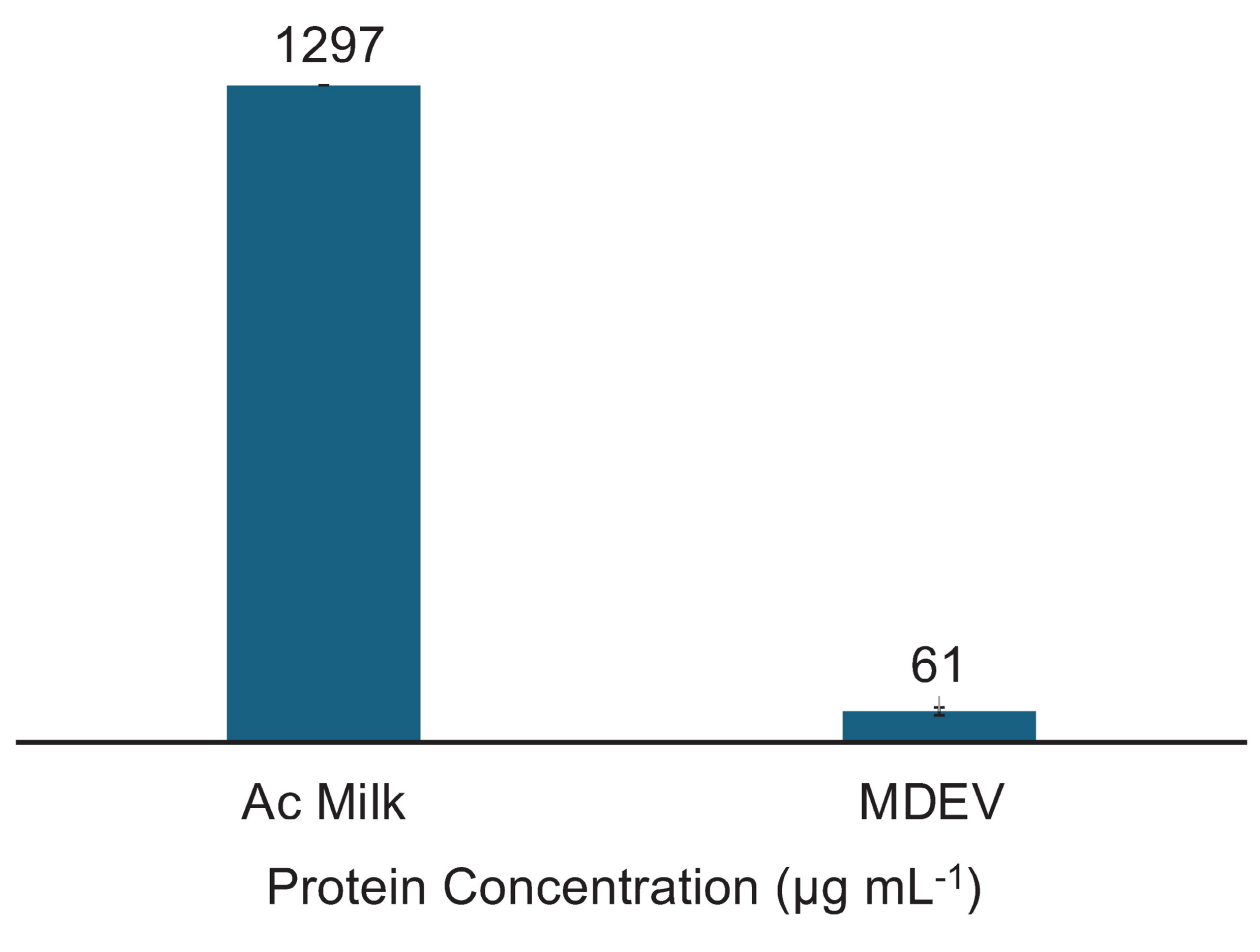
Bradford protein assay results for the protein content (µg mL^-1^) of the Ac-treated milk and the final EV isolate.

The determination of vesicle purity is a crucial factor in the downstream application of MDEVs, and perhaps the greatest challenge given the complexities of the bovine milk matrix. For these determinations, a Bradford assay was performed to obtain the concentration of protein present in the MDEV fractions from the separation. It is important to note that Bradford assay results are indicative of total protein content present in samples; the Coomassie reagent reacts with amino acids present in solution, not exclusively free proteins or vesicle related proteins; i.e., both latent proteins and those associated with the target EV surfaces register positive responses. Fractions collected from PET C-CP column isolations were stored in a 1×PBS solution after overnight evaporation of the 40% ACN at 5ºC. Each fraction was analyzed in triplicate. While the Ac-treatment of the skimmed milk should have removed a large portion of the solution phase protein in the matrix, the Ac-treated milk protein content (prior to chromatographic separation) was found to be 1297±5 µg protein mL^-1^ as depicted in **Fig. 4**. Superior to our prior MDEV experiments 42, the EV fractions here contained 61±8 µg protein mL^-1^, demonstrating a >20x decrease in total protein content versus the Ac treated milk. Using the total EV protein content present in the EV fraction and the EV concentration determined via HEK EV standard response curve (4.0×10^11^ particles mL^-1^), the average purity of the MDEV fractions from the frontal loading experiments was calculated at ~7×10^9^ EVs µg^-1^ protein, an impressive purity value from a single isolation affected in 20-min. The purity value obtained for EVs recovered from frontal loading are comparable to the previous isolations from bovine milk using PET-8 C-CP fiber columns with purities of 1.76×10^10^ particles mL^-1^ 42. It must be pointed out that this level of purity was attained with a 10-fold increase in EV recovery as reported by Vaswani et al., who achieved a purity value of 8.57×10^9^ particles µg^-1^ protein 52, 53. As mentioned previously, there is the caveat in the case of MDEVS that the high vesicular protein content skews these values relative to those from isolations from different matrices, but even so coming very near the target for human isolates.

## 4. Conclusions

As the interest in implementing MDEVs as vectors for therapeutics grows, the demand for separation methods that meet purity, concentration, and short isolation time scales does as well. A substantial challenge with using bovine milk as an EV source is the ability to achieve both high purity and efficiency yields from the matrix related fats, proteins, somatic cells, and colloidal systems within milk formed by casein micelles. Traditional separation methods such as UC, SEC, TFF, AF4, FFIEF, and polymer precipitation while successful in isolation of EVs from other matrices, struggle to obtain high purity products when isolating from the complex raw bovine milk matrix. Previously, a sample pre-treatment method of 6% Ac precipitation of milk was established for the isolation of MDEVs from small, analytical scale sample volumes (100 µL) on eight- pronged PET-8 fiber columns 42. PET-Y fibers with increased surface area (vs PET-8) were selected for maximum EV DBC based on previous characterization studies 35. Using the same Ac pre-treatment method, frontal loading of >5X the volume of pre-treated milk was loaded onto a PET-Y C-CP fiber column.

The rapid and high purity isolation of bovine milk-derived extracellular vesicles occurred in 20-min, with MDEV recoveries on the order of 4.0×10^11^ particles mL^-1^ (~1.1 X 10^12^ particles in absolute numbers) having an average diameter of ~123 nm. This concentration is on par with previous isolations affected with PET-8 C-CP columns as well, while recoveries from UC 54, TFF 55, polymer precipitation kits 15, AF4 13, FFIEF 55, and SEC 15 range from 2.78×10^9^ particles mL^-1^ to >1.2×10^12^ particles mL^-1^ 15, 54. Isolated vesicles demonstrated intact structures inherent to EVs, including intact lipid bilayers, round and unruptured structures, and population size profiles that ranged from 30-200 nm. Additionally, MDEVs yielded positive responses for lipophilic vesicle membrane dye and fluorescently labeled Abs that target tetraspanins within the MDEV lipid bilayers. The quantitative aspects of the frontal loading method demonstrated here sets the stage towards scale up of the PET-Y C-CP fiber columns for practical MDEV isolation, exhibiting high throughput (~10^12^ in 20 mins), with excellent reproducibility and purity metrics, at relatively low materials and equipment costs. Moving forward, pre-treated milk will be isolated using 2.1 mm, and larger, diameter columns, with the goal to meet throughput values of >10^14^ particles in sub-hour time scales, providing great promise for rapid bulk isolation of high quality MDEVs.

## Abbreviations

PET: polyester
C-CP: capillary-channeled polymer
HIC: hydrophobic interaction chromatography
MISEV: minimal information for studies of extracellular vesicles
PBS: phosphate buffered saline
Ac: acetic acid
EV: extracellular vesicle
MDEV: milk-derived extracellular vesicle
PEEK: polyetheretherketone
FEP: fluorinated ethylene-propylene
PES: polyethersulfone
PTFE: polytetrafluoroethylene
LDL: low-density lipoprotein
TFF: tangential field flow filtration
UC: ultracentrifugation
SEC: size exclusion chromatography
FDA: Food and Drug Administration
RNA: ribonucleic acid
DNA: deoxyribonucleic acid
HPLC: high performance liquid chromatography
AMS: ammonium sulfate
ACN: acetonitrile
DBC: dynamic binding capacity
Ab: antibody
TEM: transmission electron microscopy
HEK: human embryonic kidney
BSA: bovine serum albumin
RSD: relative standard deviation
SRM/CRMs: standard/certified reference materials
AF4: asymmetric-flow field-flow fractionation
FFIEF: free-flow isoelectric focusing
